# Abnormal Trabecular and Cortical Bone Microarchitecture in Chronic Hepatitis C Infection and Associations With Select Inflammatory Cytokines

**DOI:** 10.1093/ofid/ofaf102

**Published:** 2025-04-29

**Authors:** Erica J Weinstein, Dean M Carbonari, Craig W Newcomb, Jessie Torgersen, Shanae M Smith, Katherine L Brecker, X Sherry Liu, Jay R Kostman, Stacey Trooskin, Rebecca A Hubbard, Joshua F Baker, Babette S Zemel, Mary B Leonard, Vincent Lo Re

**Affiliations:** Division of Infectious Diseases, Department of Medicine, Perelman School of Medicine, University of Pennsylvania, Philadelphia, Pennsylvania, USA; Department of Biostatistics, Epidemiology, and Informatics, Center for Clinical Epidemiology and Biostatistics, Center for Real-World Effectiveness and Safety of Therapeutics, Perelman School of Medicine, University of Pennsylvania, Philadelphia, Pennsylvania, USA; Department of Biostatistics, Epidemiology, and Informatics, Center for Clinical Epidemiology and Biostatistics, Center for Real-World Effectiveness and Safety of Therapeutics, Perelman School of Medicine, University of Pennsylvania, Philadelphia, Pennsylvania, USA; Division of Infectious Diseases, Department of Medicine, Perelman School of Medicine, University of Pennsylvania, Philadelphia, Pennsylvania, USA; Department of Biostatistics, Epidemiology, and Informatics, Center for Clinical Epidemiology and Biostatistics, Center for Real-World Effectiveness and Safety of Therapeutics, Perelman School of Medicine, University of Pennsylvania, Philadelphia, Pennsylvania, USA; Department of Biostatistics, Epidemiology, and Informatics, Center for Clinical Epidemiology and Biostatistics, Center for Real-World Effectiveness and Safety of Therapeutics, Perelman School of Medicine, University of Pennsylvania, Philadelphia, Pennsylvania, USA; Department of Biostatistics, Epidemiology, and Informatics, Center for Clinical Epidemiology and Biostatistics, Center for Real-World Effectiveness and Safety of Therapeutics, Perelman School of Medicine, University of Pennsylvania, Philadelphia, Pennsylvania, USA; Department of Orthopedic Surgery, Perelman School of Medicine, University of Pennsylvania, Philadelphia, Pennsylvania, USA; Department of Bioengineering, University of Pennsylvania, Philadelphia, Pennsylvania, USA; Philadelphia FIGHT, Philadelphia, Pennsylvania, USA; Mazzoni Center, Philadelphia, Pennsylvania, USA; Department of Biostatistics, School of Public Health, Brown University, Providence, Rhode Island, USA; Division of Rheumatology, Department of Medicine, Perelman School of Medicine, University of Pennsylvania, Philadelphia, Pennsylvania, USA; Division of Gastroenterology, Hepatology, and Nutrition, Department of Pediatrics, Children's Hospital of Philadelphia, Perelman School of Medicine, University of Pennsylvania, Philadelphia, Pennsylvania, USA; Department of Pediatrics and Medicine, Stanford University School of Medicine, Palo Alto, California, USA; Division of Infectious Diseases, Department of Medicine, Perelman School of Medicine, University of Pennsylvania, Philadelphia, Pennsylvania, USA; Department of Biostatistics, Epidemiology, and Informatics, Center for Clinical Epidemiology and Biostatistics, Center for Real-World Effectiveness and Safety of Therapeutics, Perelman School of Medicine, University of Pennsylvania, Philadelphia, Pennsylvania, USA

**Keywords:** bone, cytokines, dual-energy x-ray absorptiometry, hepatitis C virus, peripheral quantitative computed tomography

## Abstract

**Background:**

Hepatitis C virus (HCV) infection is associated with reduced bone mineral density (BMD) and increased fracture risk. The structural underpinnings for skeletal fragility with HCV and contributions of inflammatory cytokines remain unknown. We used high-resolution peripheral quantitative computed tomography (HR-pQCT) to compare skeletal parameters by chronic HCV.

**Methods:**

We conducted a cross-sectional study among 58 participants with chronic HCV and 58 participants without HCV. Volumetric BMD and cortical dimensions of the radius and tibia were determined by HR-pQCT; visceral fat area and appendicular lean mass were assessed by whole body dual-energy x-ray absorptiometry; serum levels of tumor necrosis factor α (TNF-α), interleukin 6, and interleukin 18 were measured. Multivariable linear regression was used to estimate group differences in bone measurements and cytokines.

**Results:**

Participants with chronic HCV had lower radius trabecular volumetric BMD (−24.2 mg hydroxyapatite [HA]/cm^3^) and lower tibia trabecular volumetric BMD (−20.5 mg HA/cm^3^), cortical area (−20.9 mm^2^), and cortical thickness (−0.47 mm) than participants without HCV (all *P* < .05), independent of age, sex, visceral fat area, appendicular lean mass, and smoking. Mean log TNF-α was higher with chronic HCV (+0.1-log pg/mL; *P* < .001), but no differences in mean log interleukin 6 or interleukin 18 were observed. Higher log TNF-α was associated with lower radius trabecular volumetric BMD (−99.7 mg HA/cm^3^), lower tibia cortical volumetric BMD (−91.6 mg HA/cm^3^), and higher tibia cortical porosity (+1.39%) by HR-pQCT (all *P* < .05).

**Conclusions:**

Patients with chronic HCV had decreased trabecular volumetric BMD and cortical dimensions and higher TNF-α than individuals without infection, suggesting that HCV-associated inflammation might contribute to bone deficits.

In addition to its impact on the liver, chronic hepatitis C virus (HCV) infection exerts extrahepatic effects [1], particularly on bone, which has been referred to as “hepatic osteodystrophy” [2, 3]. Cross-sectional studies have found that chronic HCV infection is associated with low bone mineral density (BMD) by dual-energy x-ray absorptiometry (DXA) [4–7]. Cohort studies have shown that chronic HCV infection is associated with increased rates of bone fractures as compared with cases without HCV [7–9].

The mechanisms by which chronic HCV infection contributes to skeletal fragility remain unclear. Chronic HCV infection stimulates production of inflammatory cytokines, particularly tumor necrosis factor α (TNF-α), interleukin 6 (IL-6), and interleukin 18 (IL-18) [10–14]. All 3 cytokines decrease osteoblast activity and induce osteoblast apoptosis (inhibiting bone formation) [15–18], and TNF-α stimulates osteoclast activation (increasing bone resorption) [19, 20]. Together, production of these cytokines could promote low BMD. Increased prevalence of tobacco use, alcohol consumption, and other traditional osteoporosis risk factors among patients with chronic HCV infection might also contribute to low BMD and increased fracture risk [21].

There have been recent suggestions that the higher fracture risk observed in patients with chronic HCV infection is not due to low BMD alone but through additional effects on bone microarchitecture [22–24]. However, the majority of studies examining chronic HCV infection and bone health have focused on BMD as measured by DXA [25], which is a 2-dimensional method that summarizes total trabecular and cortical bone mass within the projected area. DXA is unable to distinguish deficits that occur within trabecular and cortical bone. Consequently, the structural underpinnings with respect to the impact of chronic HCV infection on cortical and trabecular bone microarchitecture are not well described. Characterizing the trabecular and cortical bone deficits and associated differences in body composition and inflammatory cytokine levels between people with and without chronic HCV could suggest mechanisms of bone loss and inform future studies of bone therapies in this at-risk and aging population. High-resolution peripheral quantitative computed tomography (HR-pQCT) is a newer 3-dimensional x-ray–based imaging technique that has the ability to discriminate between trabecular and cortical bone compartments when estimating volumetric BMD and has been shown to be highly predictive of fracture risk [26, 27].

In this study, we obtained HR-pQCT and whole body DXA scans, as well as serum concentrations of select cytokines (TNF-α, IL-6, and IL-18), among participants with and without chronic HCV to characterize the structural differences by chronic HCV status. We hypothesized that people with chronic HCV infection would have lower trabecular and cortical volumetric BMD, lower cortical area and thickness, and higher cortical porosity, as observed in other chronic inflammatory diseases [28–30], as compared with people without HCV. We also hypothesized that people with chronic HCV would have higher levels of inflammatory cytokines than those without HCV and that higher cytokine levels would be associated with greater abnormalities in bone measurements by HR-pQCT and DXA.

## METHODS

### Study Design and Setting

We performed a cross-sectional study of participants with and without chronic HCV infection who were recruited from viral hepatitis and primary care practices in the University of Pennsylvania Health System and at Philadelphia FIGHT, which are in Philadelphia, Pennsylvania. The primary care practices served as recruitment sites for participants without HCV infection.

Patients scheduled for visits were prescreened for HCV infection and approached for interest in participating by study personnel after approval by their health care provider. Interested patients were screened for potential eligibility via self-reported responses. Potentially eligible patients who provided informed consent had eligibility verified by medical record review. To promote balance in the demographics of participants between the groups, we recruited participants without HCV infection who had similar sex assigned at birth, race (Black vs not Black), and age category (18–39, 40–59, ≥60 years) to those enrolled in the group with HCV infection. Participants were recruited between 1 January 2019 and 31 July 2022. The study protocol was reviewed and approved by the University of Pennsylvania Institutional Review Board (IRB), which served as the IRB of record. The Philadelphia FIGHT IRB relied on the University of Pennsylvania IRB through a reliance agreement.

### Study Participants

Participants with chronic HCV were eligible for inclusion if they were ≥18 years of age, had detectable HCV RNA, and underwent staging of liver fibrosis within the prior 6 months. HIV coinfection was permissible if the participant had HIV RNA <200 copies/mL while undergoing a stable antiretroviral therapy regimen for ≥4 weeks prior to enrollment. Participants without HCV infection were eligible if, at enrollment, they were ≥18 years of age and HCV and HIV uninfected. All participants provided informed consent. Participants without HCV infection were confirmed to be HCV and HIV antibody negative by OraSure's OraQuick assay administered by study personnel at the study visit.

Participants from both groups were excluded if they had conditions that might affect bone: chronic kidney disease (estimated glomerular filtration rate <60 mL/min/1.73 m^2^) [31], cancer (excluding nonmelanomatous skin malignancy), conditions predisposing to malabsorption (ie, celiac disease, small bowel resection surgery, chronic diarrhea), weight loss exceeding 5% of body weight over the prior 3 months, or another chronic liver disease (ie, hepatitis B virus infection, hemochromatosis, alpha-1 antitrypsin, autoimmune hepatitis, primary sclerosing cholangitis, primary biliary cirrhosis, Wilson disease, metabolic dysfunction–associated steatotic liver disease). We also excluded patients who were pregnant (to avoid radiation from DXA) or had a history of bilateral lower leg fractures. Participants with chronic HCV were additionally excluded if they had HCV genotype 3 infection—since this promotes hepatic steatosis [32], which has been shown to be associated with low BMD [33]—or received direct-acting antiviral-based HCV therapy, since treatment might affect BMD [34]. We estimated that 58 participants per group would provide 80% power to detect a moderate effect size of a 0.5-SD difference in bone and cytokine levels between the groups, assuming a type 1 error rate of 0.05.

### Assessment of Demographic, Clinical, and Anthropometric Data

Data collected from participants included the following: age, sex assigned at birth, race, ethnicity, current smoking status, recent alcohol consumption, recent drug use, history of injection drug use, postmenopausal status (if applicable; defined as either the absence of menstrual periods for >12 months in a previously menstruating individual >45 years of age while not taking hormonal birth control or a hysterectomy or bilateral oophorectomy), history of bone fracture, and calcium and vitamin D supplement use. Alcohol consumption was determined by the 10-question Alcohol Use Disorders Identification Test [35], and drug use was determined by the 10-question Drug Abuse Screening Test [36].

Diabetes mellitus—defined by diagnosis, hemoglobin A_1c_ ≥6.5%, or random glucose ≥200 mg/dL within 12 months prior to study visit—and serum creatinine within 12 months prior to study visit were obtained from participants' medical records. For participants with chronic HCV, we also collected the earliest HCV diagnosis date, most recent HCV RNA and genotype, and stage of liver fibrosis by vibration-controlled transient elastography or HCV FibroSure test [37]. Advanced hepatic fibrosis/cirrhosis was defined as METAVIR stage F3 or F4 [37]. Body weight and height were measured in triplicate without shoes via a digital scale (Scaltronix) and stadiometer (Holtain), respectively, and the mean of each was used for analysis. Body mass index was calculated as weight in kilograms divided by height in meters squared.

### HR-pQCT Measurements

Bone measurements in the radius and tibia were obtained by HR-pQCT (Xtreme CT II; SCANCO Medical AG). The nondominant side was used unless there was a prior fracture or interfering artifact. A scout view was used to place the reference line at the distal epiphysis/end plate. Bone measurements were obtained starting at 4% of the radius length (ultradistal) and 30% of the tibia length (midshaft; each considered reference lines) for a total 110 slices representing a 9.0-mm length proximal to each reference line. Scans were analyzed for trabecular volumetric BMD (measured in mg hydroxyapatite [HA] per cubic centimeter) at the ultradistal site. Scans were analyzed for cortical volumetric BMD (mg HA per cubic centimeter), total area (square millimeter), cortical cross-sectional area (square millimeter), and cortical porosity (percentage), as well as cortical thickness, perimeter, and pore diameter (all in millimeters), at the midshaft site. Cortical volumetric BMD was assessed after exclusion of pore space and surface voxels to minimize partial volume voxels. Cortical porosity was calculated as the ratio of intracortical pore volume to total volume of the cortical compartment [38]. Cortical area, thickness, and perimeter were calculated by the direct 3-dimensional distance transformation method [39]. Mean cortical pore diameter was measured by the direct 3-dimensional method [40].

### DXA Measurements

The following were assessed with a Delphi or Horizon bone densitometer (each from Hologic): trabecular bone score (L1-L4), visceral fat area, whole body fat, and lean mass excluding bone mineral content, as well as areal BMD at the total hip, femoral neck, and lumbar spine (L1-L4). Two densitometers were employed because the Delphi was replaced by the Horizon during the conduct of the study. Quality control was monitored weekly via a phantom. Appendicular lean mass, a measure of skeletal muscle [41], and whole body fat mass were converted to appendicular lean mass index and fat mass index (both in kilograms per square meter) via height.

### Laboratory Data

Serum levels of IL-6, IL-18, and TNF-α were measured by an enzyme-linked immunosorbent assay (ProteinSimple Ella; Bio-Techne).

### Statistical Analysis

We evaluated differences in demographic and clinical characteristics, HR-pQCT and DXA measurements, and laboratory results between participants with and without chronic HCV. We then estimated mean differences (95% CIs) between the groups in bone measurements by HR-pQCT and DXA (analysis 1) and log inflammatory cytokine levels (analysis 2). We also estimated mean differences in bone measurements by HR-pQCT and DXA associated with a 1-log increase in cytokine level (analysis 3). We developed directed acyclic graphs to identify relevant confounders, mediators, and colliders in the relationship between exposure and outcome in each analysis to determine covariates for adjustment in these analyses (Supplementary Figure 1).

For analysis 1, we used multivariable linear regression to estimate the mean difference (95% CI) in HR-pQCT and DXA bone measurements between participants with and without chronic HCV infection, after adjustment for age (continuous), sex, appendicular lean mass index by DXA (continuous), visceral fat area by DXA (continuous), and current smoking. We chose to adjust for appendicular lean mass index and visceral fat area because prior data have shown that bone quality is independently associated with muscle mass [42, 43] and visceral fat mass (Supplementary Figure 1A) [44–53]. Since the initial DXA scanner was replaced during the study, analyses of DXA outcomes were additionally adjusted for densitometer. To assess the robustness of the results, we repeated our analyses replacing visceral fat area with fat mass index (continuous).

To explore the effects of HIV/HCV coinfection on bone outcomes, we repeated analysis 1, stratifying the HCV group by HIV coinfection status. We then used multivariable linear regression to estimate the mean difference (95% CI) in HR-pQCT and DXA bone measurements between the uninfected control group and the participants with (1) HIV/HCV coinfection and (2) HCV alone, after adjustment for age, sex assigned at birth, visceral fat area by DXA, appendicular lean mass index by DXA, and current smoking. Moreover, in a post hoc analysis, we repeated analysis 1 additionally adjusting for TNF-α level. Observing attenuated differences in HR-pQCT and DXA bone measurements between participants with HCV infection and uninfected controls after further adjustment for TNF-α would provide supportive evidence that inflammation is in the causal pathway between HCV infection and bone deficits.

For analysis 2, we used multivariable linear regression to estimate the mean difference (95% CI) in log IL-6, IL-18, and TNF-α levels between participants with and without HCV, after adjustment for age (continuous), sex assigned at birth, visceral fat area by DXA (continuous), and smoking. We adjusted for visceral fat because such fat depots are known to contribute to secretion of inflammatory cytokines [54–58]. We did not adjust for appendicular lean mass index, since it was deemed a collider in the relationship between chronic HCV and the cytokines (Supplementary Figure 1B). We log transformed cytokine levels because their relationship with continuous covariates was more approximately linear after log transformation. Analyses were repeated replacing visceral fat area with fat mass index (continuous).

For analysis 3, we used multivariable linear regression to estimate the mean difference (95% CI) in HR-pQCT and DXA bone measurements per log cytokine level among all participants. Analyses were adjusted for age (continuous), sex assigned at birth, visceral fat area (continuous), appendicular lean mass index (continuous), smoking, and HCV infection. Analyses of DXA outcomes were additionally adjusted for densitometer. Analyses were again repeated replacing visceral fat area with fat mass index.

The linearity of relationships between outcomes and continuous predictor variables in all analyses was confirmed by examination of scatter plots. Analyses were conducted in SAS version 9.4 (SAS Institute Inc).

## RESULTS

### Participant Characteristics

**Figure 1.**
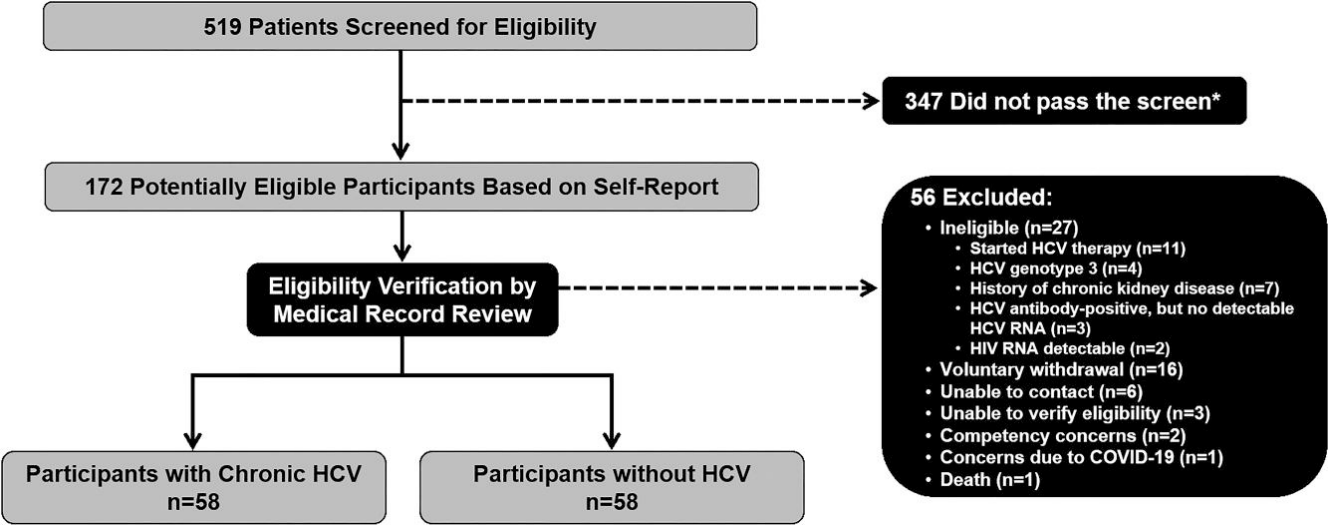
Cohort selection flow. HCV, hepatitis C virus. *See Supplementary Methods 1 for a list of screening questions.

Among 519 patients screened for eligibility, 172 were deemed potentially eligible based on self-report (Figure 1). Of these 172 patients, 56 were excluded (reasons for exclusion are reported in Figure 1), yielding 116 participants who completed the study visit: 58 with chronic HCV and 58 without HCV infection. Twelve (21%) participants with chronic HCV also had HIV coinfection with undetectable HIV RNA on antiretroviral therapy: 11 received a regimen containing tenofovir alafenamide; 1 received a regimen of dolutegravir/abacavir/lamivudine. Participants with chronic HCV were more likely to currently smoke and report drug use within the past year (Table 1). No other significant differences in demographic or clinical characteristics between the groups were observed. Thirteen participants with chronic HCV had advanced hepatic fibrosis/cirrhosis based on liver fibrosis staging.

**Table 1.** Characteristics of Participants Who Completed Enrollment Study Visit, by Chronic Hepatitis C Virus Status

Characteristic	Participants With HCV (n = 58)	Participants Without HCV (n = 58)	*P* Value
Recruitment site			
University of Pennsylvania Health System	41 (70.7)	52 (89.7)	
Philadelphia FIGHT	17 (29.3)	0 (0.0)	
Referral	0 (0.0)	6 (10.3)	
Age, y, median (IQR)	57 (42–64)	54 (36–63)	.267
Missing	0 (0.0)	0 (0.0)	
Male sex	42 (72.4)	36 (62.1)	.235
Missing	0 (0.0)	0 (0.0)	
Race			.333
Asian	0 (0)	2 (3.4)	
Black or African American	38 (65.5)	34 (58.6)	
White	18 (31.0)	19 (32.8)	
Others	2 (3.4)	3 (5.2)	
Missing	0 (0.0)	0 (0.0)	
Hispanic ethnicity	4 (6.9)	4 (6.9)	>.99
Missing	0 (0.0)	0 (0.0)	
Body mass index, kg/m^2^			
Median (IQR)	27 (24–31)	28 (25–33)	.139
Category			.309
Underweight: <18.50	0 (0)	1 (1.7)	
Normal: 18.50–24.99	18 (31.0)	14 (24.1)	
Overweight: 25.00–29.99	22 (37.9)	17 (29.3)	
Obesity: ≥30.00	18 (31.0)	26 (44.8)	
Missing	0 (0.0)	0 (0.0)	
Diabetes mellitus^a^	15 (25.9)	12 (20.7)	.510
Missing	0 (0.0)	0 (0.0)	
Serum creatinine, mg/dL, median (IQR)^b^	0.93 (0.81–1.05)	0.96 (0.83–1.07)	.478
Missing	2 (3.4)	17 (29.3)	**<**.**001**
History of bone fracture	33 (56.9)	23 (39.7)	.063
Missing	0 (0.0)	0 (0.0)	
Calcium supplement use	7 (12.1)	7 (12.1)	>.99
Missing	0 (0.0)	0 (0.0)	
Vitamin D supplement use	11 (19.0)	17 (29.3)	.193
Missing	0 (0.0)	0 (0.0)	
Postmenopausal: females only^c^	8/16 (50.0)	9/22 (40.9)	.578
Missing	0 (0.0)	0 (0.0)	
Smoking status			**<**.**001**
Never	5 (8.6)	34 (58.6)	
Former	10 (17.2)	10 (17.2)	
Current	43 (74.1)	14 (24.1)	
Missing	0 (0.0)	0 (0.0)	…
Smoking pack-years, median (IQR)^d^	8.00 (4.25–12.00)	6.50 (2.15–13.50)	.815
Missing	0 (0.0)	0 (0.0)	
Self-reported history of injection drug use	31 (53.4)	2 (3.4)	**<.001**
Time since HCV diagnosis, y^e^			
Median (IQR)	3 (1–13)		
Mean (SD)	7 (9)		
<5	34 (58.6)		
5–10	8 (13.8)		
11–20	10 (17.2)		
21–40	6 (10.3)		
Alcohol use in past year: AUDIT score			
Median (IQR)	1 (0–4)	2 (1–4)	.103
Category			.942
<8: low risk	52 (89.7)	53 (91.4)	
8–15: risky/hazardous	5 (8.6)	4 (6.9)	
16–19: high risk	0 (0.0)	0 (0.0)	
≥20: highest risk	1 (1.7)	1 (1.7)	
Missing	0 (0.0)	0 (0.0)	
Drug use in past year: DAST-10 score			
Median (IQR)	1 (0–6)	0 (0–1)	.**005**
Category			**<**.**001**
0: none	27 (46.6)	42 (72.4)	
1 or 2: low	8 (13.8)	15 (25.9)	
3–5: moderate	6 (10.3)	1 (1.7)	
6–8: substantial	11 (19.0)	0 (0)	
9 or 10: severe	6 (10.3)	0 (0)	
Missing	0 (0.0)	0 (0.0)	
Log cytokine levels, pg/mL, mean (SD)			
Interleukin 6	0.5 (0.41)	0.4 (0.34)	.072
Interleukin 18	2.4 (0.25)	2.3 (0.20)	.**002**
Tumor necrosis factor α	1.1 (0.12)	1.0 (0.11)	**<**.**001**
Missing	9 (15.5)	1 (1.7)	.**008**

Results are reported as No. (%) unless otherwise noted. Bold indicates *P* < .05.

Abbreviations: AUDIT, Alcohol Use Disorders Identification Test; DAST-10, 10-question Drug Abuse Screening Test; HCV, hepatitis C virus.

^a^Diabetes mellitus was defined as the presence of any of the following: diagnosis of diabetes in the medical record, hemoglobin A_1c_ ≥6.5%, or random glucose ≥200 mg/dL.

^b^Serum creatinine levels from the prior 12 months, if available, were collected from the medical record.

^c^Postmenopausal status among participants assigned female at birth defined as either the absence of menstrual periods for 12 months in a previously menstruating individual >45 years of age while taking hormonal birth control or status post hysterectomy or bilateral oophorectomy.

^d^For current smokers, smoking pack-years were calculated as the average number of cigarettes smoked per day divided by 20, then multiplied by the number of years that the person has smoked.

^e^Time since initial HCV diagnosis based on the self-reported year of initial HCV diagnosis.

### Group Differences in HR-pQCT and DXA Measurements

Based on HR-pQCT of the radius, participants with chronic HCV had lower trabecular and cortical volumetric BMD but higher total area and cortical porosity when compared with participants without HCV (Table 2). With HR-pQCT of the tibia, only trabecular volumetric BMD was lower among participants with chronic HCV. No differences in other HR-pQCT tibial measurements were observed between the groups.

**Table 2.** Mean HR-pQCT and DXA Measurements in Participants With and Without Hepatitis C Virus

Measurement	Participants With HCV (n = 58)	Participants Without HCV (n = 58)
HR-pQCT of Radius^a^		
Trabecular volumetric BMD,^b^ mg HA/cm^3^	153.7 (45.74)	179.4 (42.36)
Total area, mm^2^	131.3 (23.12)	118.3 (22.40)
Cortical area, mm^2^	101.5 (18.43)	97.0 (17.21)
Cortical volumetric BMD, mg HA/cm^3^	1071.8 (33.35)	1087.8 (26.62)
Cortical porosity, %	0.60 (0.55)	0.40 (0.31)
Cortical perimeter, mm	45.3 (4.48)	43.5 (6.83)
Cortical thickness, mm	3.4 (0.54)	3.5 (0.43)
Cortical pore diameter, mm	0.175 (0.03)	0.167 (0.03)
HR-pQCT of Tibia^c^		
Trabecular volumetric BMD,^b^ mg HA/cm^3^	147.9 (53.01)	174.4 (33.91)
Total area, mm^2^	429.5 (66.13)	425.7 (69.48)
Cortical area, mm^2^	300.3 (52.78)	310.6 (57.81)
Cortical volumetric BMD, mg HA/cm^3^	1007.9 (34.27)	1012.8 (35.04)
Cortical porosity, %	0.96 (0.70)	0.87 (0.72)
Cortical perimeter, mm	83.7 (6.57)	83.3 (7.43)
Cortical thickness, mm	5.8 (1.02)	6.1 (1.04)
Cortical pore diameter, mm	0.207 (0.04)	0.224 (0.06)
DXA		
Total hip BMD,^d^ g/cm^2^	0.98 (0.17)	1.07 (0.17)
Femoral neck BMD,^d^ g/cm^2^	0.87 (0.17)	0.92 (0.18)
Lumbar spine BMD,^e^ g/cm^2^	1.08 (0.21)	1.16 (0.19)
Trabecular bone score^f^	1.35 (0.13)	1.38 (0.13)
Appendicular lean mass index,^g^ kg/m^2^	7.99 (1.28)	8.06 (1.44)
Whole body fat mass index,^g^ kg/m^2^	8.65 (3.87)	9.73 (4.91)
Visceral fat area,^g^ cm^2^	89.69 (49.88)	108.70 (66.65)

Results are reported as mean (SD).

Abbreviations: BMD, bone mineral density; DXA, dual-energy x-ray absorptiometry; HA, hydroxyapatite; HCV, hepatitis C virus; HR-pQCT, high-resolution peripheral quantitative computed tomography.

^a^No. (%) of missing HR-pQCT measurements at the radius in participants with vs without HCV: 5 (8.6%) vs 3 (5.2%; *P* > .05), except for trabecular volumetric BMD, which was 5 (8.6%) vs 1 (1.7%; *P* > .05).

^b^Trabecular volumetric BMD measurement was conducted at the ultradistal location; measurement for all the HR-pQCT categories thereafter occurred at the midshaft location.

^c^No. (%) of missing HR-pQCT measurements at the tibia in participants with vs without HCV: 7 (12.1%) vs 7 (12.1%; *P* > .05), except for trabecular volumetric BMD, which was 7 (12.1%) vs 4 (6.9%; *P* > .05).

^d^No. (%) of missing DXA measurement in participants with vs without HCV: 3 (5.2%) vs 3 (5.2%; *P* > .05).

^e^No. (%) of missing DXA measurement in participants with vs without HCV: 4 (6.9%) vs 2 (3.4%; *P* > .05).

^f^No. (%) of missing DXA measurement in participants with vs without HCV: 1 (1.7%) vs 1 (1.7%; *P* > .05).

^g^No. (%) of missing DXA measurement in participants with vs without HCV: 3 (5.2%) vs 2 (3.4%; *P* > .05).

Among participants with HCV, 76% used the Delphi bone densitometer and 24% used the Horizon. Among participants without HCV, 23% used the Delphi and 77% used the Horizon. Total hip BMD and lumbar spine BMD by DXA were lower in participants with chronic HCV, but there were no significant differences between the groups in the other DXA measurements (Table 2).

After adjustment for age, sex assigned at birth, visceral fat area, appendicular lean mass index, and current smoking, participants with chronic HCV had lower trabecular volumetric BMD (−24.2 mg HA/cm^3^) of the radius and lower trabecular volumetric BMD (−20.5 mg HA/cm^3^), cortical area (−20.9 mm^2^), and cortical thickness (−0.47 mm) of the tibia by HR-pQCT as compared with participants without HCV (all *P* < .05; Table 3). No differences in total hip, femoral neck, lumbar spine, or trabecular bone score by DXA were observed between the groups in models adjusted for age, sex, visceral fat area, appendicular lean mass index, smoking, and densitometer. Results were similar when visceral fat area was replaced with fat mass index (Supplementary Table 1).

**Table 3.** Mean Differences (95% CIs) in Bone Measurements Between Participants With and Without Hepatitis C Virus Infection

	Unadjusted		Adjusted^a^	
Bone Measurement	Mean Difference (95% CI)	*P* Value	Mean Difference (95% CI)	*P* Value
HR-pQCT of Radius				
Trabecular volumetric BMD,^b^ mg HA/cm^3^	−25.7 (−42.4, −9.06)	.**003**	−24.2 (−42.7, −5.72)	.**011**
Total area, mm^2^	12.98 (4.298, 21.66)	.**004**	3.977 (−4.61, 12.57)	.360
Cortical volumetric BMD, mg HA/cm^3^	−16.0 (−27.5, −4.51)	.**007**	−8.32 (−21.6, 4.975)	.217
Cortical area, mm^2^	4.532 (−2.27, 11.33)	.189	−0.662 (−6.56, 5.234)	.824
Cortical porosity, %	0.194 (.025, .364)	.**025**	0.101 (−.057, .258)	.207
Mean cortical perimeter, mm	1.768 (−.445, 3.981)	.116	0.734 (−1.73, 3.202)	.556
Mean cortical thickness, mm	−0.110 (−.296, .075)	.242	−0.124 (−.304, .057)	.177
Mean cortical pore diameter, mm	0.008 (−.004, .020)	.184	0.001 (−.013, .014)	.933
HR-pQCT of Tibia				
Trabecular volumetric BMD,^b^ mg HA/cm^3^	−26.5 (−43.6, −9.35)	.**003**	−20.5 (−40.9, −.078)	.**049**
Total area, mm^2^	3.825 (−22.8, 30.47)	.776	−10.3 (−37.1, 16.44)	.446
Cortical volumetric BMD, mg HA/cm^3^	−4.92 (−18.5, 8.694)	.475	−2.19 (−18.8, 14.42)	.794
Cortical area, mm^2^	−10.4 (−32.1, 11.37)	.346	−20.9 (−38.3, −3.48)	.**019**
Cortical porosity, %	0.086 (−.192, .364)	.540	0.051 (−.270, .372)	.753
Mean cortical perimeter, mm	0.380 (−2.37, 3.135)	.785	−1.02 (−3.69, 1.641)	.447
Mean cortical thickness, mm	−0.336 (−.740, .068)	.102	−0.471 (−.818, −.125)	.**008**
Mean cortical pore diameter, mm	−0.018 (−.038, .003)	.086	−0.013 (−.037, .011)	.277
DXA^c^				
Total hip BMD, g/cm^2^	−0.070 (−.145, .005)	.069	−0.028 (−.109, .053)	.492
Femoral neck BMD, g/cm^2^	−0.045 (−.123, .033)	.256	−0.011 (−.098, .076)	.800
Lumbar spine BMD, g/cm^2^	−0.038 (−.129, .052)	.400	−0.024 (−.128, .079)	.641
Trabecular bone score	−0.034 (−.092, .024)	.244	−0.020 (−.080, .039)	.496

Bold indicates *P* < .05.

Abbreviations: BMD, bone mineral density; DXA, dual-energy x-ray absorptiometry; HA, hydroxyapatite; HCV, hepatitis C virus; HR-pQCT, high-resolution peripheral quantitative computed tomography.

^a^Adjusted for age, sex, appendicular lean mass index, visceral fat area, and current smoking.

^b^Trabecular volumetric BMD measurement was conducted at the ultradistal location; measurement for all the HR-pQCT categories thereafter occurred at the midshaft location.

^c^All analyses additionally adjusted for DXA machine since the machine was changed during the course of the study.

When we stratified the HCV group by HIV status and repeated the analysis, we observed that bone deficits at the radius and tibia were larger in magnitude among participants with HIV/HCV coinfection (n = 12) than for those with chronic HCV alone (n = 46). Participants with HIV/HCV coinfection had lower trabecular volumetric BMD (−40.4 mg HA/cm^3^) and cortical thickness (−0.35 mm) of the radius and lower trabecular volumetric BMD (−55.1 mg HA/cm^3^), cortical area (−28.5 mm^2^), and cortical thickness (−0.80 mm) of the tibia by HR-pQCT as compared with the uninfected control group (all *P* < .05; Table 4), after adjustment for age, sex, visceral fat area by DXA, appendicular lean mass index by DXA, and current smoking.

**Table 4.** Mean Differences (95% CIs) in Bone Measurements Between Participants With and Without HIV and Hepatitis C Virus Infection

	Primary Analysis		Secondary Analysis			
	HIV/HCV and HCV Combined (n = 58)		HIV/HCV Only (n = 12)		HCV Only (n = 46)	
Bone Measurement	Mean Difference (95% CI)	*P* Value	Mean Difference (95% CI)	P Value	Mean Difference (95% CI)	*P* Value
HR-pQCT of Radius						
Trabecular vBMD,^a^ mg HA/cm^3^	−24.2 (−42.7, −5.72)	.**011**	−40.4 (−69.3, −11.6)	.**0065**	−18.9 (−38.7, .832)	.0602
Total area, mm^2^	3.977 (−4.61, 12.57)	.360	4.735 (−8.59, 18.06)	.4822	3.728 (−5.53, 12.98)	.4258
Cortical vBMD, mg HA/cm^3^	−8.32 (−21.6, 4.975)	.217	−17.2 (−37.7, 3.299)	.0991	−5.40 (−19.6, 8.824)	.4527
Cortical area, mm^2^	−0.662 (−6.56, 5.234)	.824	−4.28 (−13.4, 4.816)	.3526	0.526 (−5.79, 6.844)	.8691
Cortical porosity, %	0.101 (−.057, .258)	.207	0.213 (−.029, .456)	.0836	0.064 (−.105, .232)	.4548
Mean cortical perimeter, mm	0.734 (−1.73, 3.202)	.556	1.013 (−2.81, 4.841)	.6004	0.642 (−2.02, 3.301)	.6326
Mean cortical thickness, mm	−0.124 (−.304, .057)	.177	−0.346 (−.619, −.073)	.**0136**	−0.051 (−.240, .139)	.5975
Mean cortical pore diameter, mm	0.001 (−.013, .014)	.933	0.002 (−.019, .023)	.8706	0.000 (−.015, .015)	.9788
HR-pQCT of Tibia						
Trabecular vBMD,^a^ mg HA/cm^3^	−20.5 (−40.9, −.078)	.**049**	−55.1 (−84.3, −25.9)	.**0003**	−7.77 (−28.8, 13.31)	.4662
Total area, mm^2^	−10.3 (−37.1, 16.44)	.446	−0.546 (−40.2, 39.10)	.9783	−14.0 (−43.0, 15.01)	.3404
Cortical vBMD, mg HA/cm^3^	−2.19 (−18.8, 14.42)	.794	−12.8 (−37.2, 11.72)	.3033	1.784 (−16.1, 19.69)	.8435
Cortical area, mm^2^	−20.9 (−38.3, −3.48)	.**019**	−28.5 (−54.3, −2.74)	.**0305**	−18.0 (−36.9, .820)	.0606
Cortical porosity, %	0.051 (−.270, .372)	.753	0.149 (−.327, .626)	.5349	0.014 (−.334, .362)	.9359
Mean cortical perimeter, mm	−1.02 (−3.69, 1.641)	.447	−0.282 (−4.23, 3.670)	.8874	−1.30 (−4.19, 1.588)	.3732
Mean cortical thickness, mm	−0.471 (−.818, −.125)	.**008**	−0.798 (−1.30, −.292)	.**0023**	−0.349 (−.719, .021)	.0645
Mean cortical pore diameter, mm	−0.013 (−.037, .011)	.277	−0.018 (−.053, .018)	.3263	−0.011 (−.037, .014)	.3817
DXA^b^						
Total hip BMD, g/cm^2^	−0.028 (−.109, .053)	.492	−0.071 (−.187, .044)	.2236	−0.017 (−.101, .066)	.6839
Femoral neck BMD, g/cm^2^	−0.011 (−.098, .076)	.800	−0.058 (−.183, .067)	.3605	0.001 (−.090, .091)	.9901
Lumbar spine BMD, g/cm^2^	−0.024 (−.128, .079)	.641	−0.115 (−.261, .032)	.1233	−0.002 (−.107, .104)	.9776
Trabecular bone score	−0.020 (−.080, .039)	.496	−0.045 (−.129, .040)	.2999	−0.014 (−.076, .047)	.6465

All models are adjusted for age, sex, appendicular lean mass index, visceral fat area, and current smoking. Bold indicates *P* < .05.

Abbreviations: BMD, bone mineral density; DXA, dual-energy x-ray absorptiometry; HA, hydroxyapatite; HCV, hepatitis C virus; HR-pQCT, high-resolution peripheral quantitative computed tomography; vBMD, volumetric bone mineral density.

^a^Trabecular vBMD measurement was conducted at the ultradistal location; measurement for all the HR-pQCT categories thereafter occurred at the midshaft location.

^b^All analyses additionally adjusted for DXA machine since the machine was changed during the course of the study.

When we repeated the analysis additionally adjusting for TNF-α level, we found that the group differences in HR-pQCT and DXA bone measurements were diminished and that the associations between chronic HCV infection and bone measurements were attenuated (Supplementary Table 2).

### Group Differences in Inflammatory Cytokines

Mean log IL-18 and TNF-α levels were significantly higher in participants with chronic HCV than in participants without HCV (Table 1). There were no differences in mean log IL-6 levels between the groups.

After adjustment for age, sex assigned at birth, visceral fat area, and current smoking, mean log TNF-α levels remained higher among participants with chronic HCV (+0.1-log pg/mL, *P* < .001), but there were no statistically significant differences in mean log IL-6 or IL-18 levels between the groups (Table 5). Similar results were observed when visceral fat area was replaced with fat mass index (Supplementary Table 3).

**Table 5.** Mean Differences (95% CIs) in Log Levels of Cytokines Between Participants With and Without Hepatitis C Virus Infection

	Unadjusted		Adjusted^a^	
Log Cytokine, pg/mL	Mean Difference (95% CI)	*P* Value	Mean Difference (95% CI)	*P* Value
Interleukin 6	0.134 (−.012, .280)	.072	0.161 (−.000, .322)	.050
Interleukin 18	0.138 (.051, .224)	.**002**	0.075 (−.025, .176)	.140
TNF-α	0.114 (.069, .158)	**<**.**001**	0.100 (.044, .152)	**<**.**001**

Bold indicates *P* < .05.

Abbreviation: TNF-α, tumor necrosis factor α.

^a^Adjusted for age, sex, visceral fat area, and current smoking.

### Associations Between Cytokine Levels and HR-pQCT and DXA Bone Measurements

In multivariable linear regression analyses adjusting for age, sex, visceral fat area, appendicular lean mass index, current smoking, and HCV infection, higher log TNF-α levels were associated with lower radius trabecular volumetric BMD (−99.7 mg HA/cm^3^), lower tibia cortical volumetric BMD (−91.6 mg HA/cm^3^), and higher tibia cortical porosity (+1.39%) by HR-pQCT, as well as lower trabecular bone score (−0.453) by DXA (all *P* < .05; Table 6). Higher log IL-6 levels were associated with higher tibia cortical porosity (+0.48%) by HR-pQCT (*P* < .05). Higher log IL-18 levels were associated with lower radius trabecular volumetric BMD (−42.5 mg HA/cm^3^) by HR-pQCT (*P* < .05). Results were similar after substituting fat mass index for visceral fat area (Supplementary Table 4).

**Table 6.** Adjusted Mean Differences (95% CIs) in HR-pQCT and DXA Bone Measurements per 1.0-Log Increase in Specified Cytokine Level Among All Participants

	Mean Difference^a^ (95% CI) in Bone Measurement per 1.0-Log Increase in Cytokine					
Bone Measurement	Log IL-6	*P* Value	Log IL-18	*P* Value	Log TNF-α	*P* Value
HR-pQCT of Radius						
Trabecular vBMD,^b^ mg HA/cm^3^	11.37 (−15.3, 38.04)	.399	−42.5 (−83.8, −1.33)	.**043**	−99.7 (−179, −20.8)	.**014**
Total area, mm^2^	0.076 (−11.8, 11.96)	.999	−4.51 (−23.4, 14.38)	.636	−0.922 (−37.1, 35.21)	.960
Cortical vBMD, mg HA/cm^3^	3.380 (−13.4, 20.20)	.691	7.341 (−19.6, 34.29)	.590	2.623 (−48.6, 53.83)	.919
Cortical area, mm^2^	2.483 (−5.55, 10.52)	.541	−7.53 (−20.2, 5.191)	.243	−2.88 (−27.4, 21.62)	.816
Cortical porosity, %	0.132 (−.082, .347)	.224	−0.119 (−.463, .224)	.492	0.405 (−.247, 1.057)	.220
Mean cortical perimeter, mm	0.143 (−3.28, 3.569)	.934	−1.35 (−6.80, 4.094)	.623	2.809 (−7.62, 13.24)	.594
Mean cortical thickness, mm	0.208 (−.027, .442)	.082	−0.175 (−.553, .203)	.360	0.013 (−.714, .741)	.971
Mean cortical pore diameter, mm	0.012 (−.006, .030)	.195	−0.009 (−.039, .020)	.535	0.025 (−.030, .080)	.157
HR-pQCT of Tibia						
Trabecular vBMD,^b^ mg HA/cm^3^	10.77 (−16.7, 38.27)	.438	−41.5 (−84.0, .899)	.055	−49.5 (−133, 34.00)	.242
Total area, mm^2^	11.42 (−24.5, 47.33)	.529	9.002 (−47.6, 65.56)	.752	14.47 (−97.0, 126.0)	.797
Cortical vBMD, mg HA/cm^3^	−11.1 (−31.5, 9.331)	.283	−18.5 (−50.9, 13.91)	.260	−91.6 (−152, −31.1)	.**004**
Cortical area, mm^2^	2.225 (−21.9, 26.35)	.855	7.241 (−30.9, 45.40)	.707	−19.7 (−94.6, 55.17)	.602
Cortical porosity, %	0.477 (.084, .869)	.**018**	0.248 (−.390, .886)	.441	1.388 (.172, 2.605)	.**026**
Mean cortical perimeter, mm	1.566 (−1.94, 5.076)	.377	2.851 (−2.67, 8.375)	.308	3.994 (−6.90, 14.89)	.468
Mean cortical thickness, mm	−0.130 (−.595, .335)	.578	0.008 (−.734, .749)	.983	−0.679 (−2.13, .770)	.354
Mean cortical pore diameter, mm	0.017 (−.016, .050)	.316	−0.000 (−.053, .052)	.986	−0.009 (−.111, .093)	.862
DXA^c^						
Total hip BMD, g/cm^2^	0.064 (−.033, .162)	.192	−0.066 (−.219, .088)	.398	−0.272 (−.567, .023)	.070
Femoral neck BMD, g/cm^2^	0.095 (−.009, .199)	.072	−0.064 (−.230, .101)	.443	−0.292 (−.611, .027)	.072
Lumbar spine BMD, g/cm^2^	0.052 (−.071, .176)	.401	−0.062 (−.261, .137)	.539	−0.322 (−.697, .054)	.092
Trabecular bone score	−0.084 (−.152, −.015)	.**017**	−0.020 (−.132, .092)	.723	−0.245 (−.456, −.035)	.**023**

Bone measurements are adjusted for age, sex assigned at birth, appendicular lean mass index, visceral fat area, current smoking, and chronic hepatitis C virus infection. Bold indicates *P* < .05.

Abbreviations: BMD, bone mineral density; HA, hydroxyapatite; IL, interleukin; DXA, dual-energy x-ray absorptiometry; HA, hydroxyapatite; HCV, hepatitis C virus; HR-pQCT, high-resolution peripheral quantitative computed tomography; TNF-α, tumor necrosis factor α; vBMD, volumetric bone mineral density.

^a^Mean difference was adjusted for age, sex assigned at birth, appendicular lean mass index, fat mass index, current smoking, and chronic HCV infection.

^b^Trabecular vBMD measurement was conducted at the ultradistal location; measurement for all the HR-pQCT categories thereafter occurred at the midshaft location.

^c^All analyses additionally adjusted for DXA machine since the machine was changed during the course of the study.

## DISCUSSION

Our study found that participants with chronic HCV infection had substantially inferior bone microarchitecture when compared with participants without HCV, including lower radius trabecular volumetric BMD and lower tibia trabecular volumetric BMD, cortical area, and cortical thickness by HR-pQCT. Bone deficits were larger for the subgroup of participants with chronic HCV who also had HIV coinfection. Observed differences in bone measurements were independent of age, sex, visceral fat area, appendicular lean mass, and smoking. Mean log TNF-α levels were higher in participants with chronic HCV after adjustment for age, sex assigned at birth, visceral fat area, and smoking, but no differences in mean log IL-6 or IL-18 levels were observed between the groups. Higher log TNF-α levels were associated with lower radius trabecular volumetric BMD, lower tibia cortical volumetric BMD, and higher tibia cortical porosity by HR-pQCT, as well as lower trabecular bone score by DXA. Additionally, higher log IL-6 levels were associated with higher tibia cortical porosity by HR-pQCT, and higher log IL-18 levels were associated with lower radius trabecular volumetric BMD by HR-pQCT. These findings suggest that chronic HCV infection adversely affects bone quality and that these cytokines play a potentially important role in the mechanism of these bone deficits.

The lower radius trabecular volumetric BMD and lower tibia trabecular volumetric BMD, cortical area, and thickness in participants with chronic HCV as compared with participants without HCV may contribute to increased fracture risk in this group, and these findings support the hypothesis that HCV-mediated chronic inflammation contributes to the structural bone deficits observed in this group. One recent cohort study found that rates of bone fractures increased 10% to 60% for each 1.0-SD deficit in HR-pQCT measurement of the tibia and radius [59]. Our study observed deficits of approximately 0.5 SD in HR-pQCT outcomes among participants with HCV infection as compared with uninfected controls (Tables 3 and 4). Studies of people with inflammatory bowel disease [28, 29] and rheumatoid arthritis [30] have demonstrated a similar pattern of trabecular bone loss and decreased cortical dimensions. The higher levels of TNF-α in the chronic HCV group—along with associations between higher log TNF-α levels and lower trabecular bone score, lower tibia cortical volumetric BMD, and higher tibia cortical porosity—further suggest the contribution of HCV-related inflammation. Additionally, associations between chronic HCV and bone measurements were attenuated after additional adjustment for TNF-α levels, providing further evidence that systemic inflammation may be driving bone deficits in people with chronic HCV infection. TNF-α can reduce bone formation by inhibiting osteoblastogenesis and inducing osteoblast apoptosis [15, 18]. TNF-α can also promote trabecular and cortical bone resorption by inducing expression of RANKL (receptor activator of nuclear factor kappa B ligand), which stimulates osteoclasts to resorb bone [19, 20].

Our results extend the observations on bone microarchitecture of prior pQCT studies in people with chronic HCV [22, 24]. One cross-sectional study among 50 women with HIV/HCV coinfection, 51 women with HCV monoinfection, and 50 women with HIV monoinfection measured tibial volumetric BMD and cortical dimensions by pQCT and generated race-specific *Z* scores for age using 263 female reference participants without HIV or liver disease [22]. Participants with coinfection had lower mean trabecular volumetric BMD, cortical volumetric BMD, cortical area, and cortical thickness *Z* scores than reference participants (all *P* < .001). The smaller cortical dimensions were due to greater mean endosteal circumference and comparable periosteal circumference *Z* scores. A separate study measured volumetric BMD of the distal radius and tibia by HR-pQCT in 37 women with HIV/HCV coinfection and 119 postmenopausal women with HIV infection alone [24]. After adjustment for body mass index, smoking, and prior AIDS diagnosis, tibial total volumetric BMD by HR-pQCT was lower in the HIV/HCV group (114.2 vs 137.1 mg HA/cm^3^, *P* = .03).

Our study had several limitations. First, the cross-sectional design did not allow us to evaluate changes in HR-pQCT measurements and other osteoporosis risk factors over time. Second, our analyses did not explore the impact of the duration of chronic HCV (since the date of HCV infection is challenging to ascertain), poor nutrition, or certain comorbidities (eg, heart failure, thyroid disease). Furthermore, use of opioids has been associated with lower BMD [60, 61], but we did not collect data on use of prescribed or illicit opioids. These factors might be important contributors to bone deficits in this population. Third, although well-controlled HIV infection and advanced hepatic fibrosis/cirrhosis could play a role in bone deficits and cytokine levels, we had too few participants with these comorbidities to permit examination of their associations with bone deficits and cytokine levels. Finally, our study is not generalizable to patients with chronic HCV who received treatment with direct-acting antiviral therapy. Future studies should examine HR-pQCT measurements in this group.

Our study had a number of strengths. We assessed bone microarchitecture using a state-of-the-art HR-pQCT machine, which provides more accurate quantification of trabecular and cortical structure than either DXA or conventional pQCT. Notably, no differences in total hip, femoral neck, or lumbar spine by DXA were observed between participants with and without chronic HCV infection after adjustment (Table 3), but HR-pQCT was able to detect substantially inferior bone microarchitecture in participants with chronic HCV infection, highlighting its ability to provide valuable insights into the effects of HCV infection on discrete components of trabecular and cortical bone. We measured levels of potentially important chronic HCV–induced cytokines that affect osteoblasts and osteoclasts to estimate associations with observed bone deficits. We also assessed and controlled for important potential confounding variables, particularly visceral fat, whole body fat mass, appendicular lean mass, and smoking status.

In conclusion, participants with chronic HCV infection had inferior trabecular volumetric BMD and cortical dimensions along with higher TNF-α levels when compared with participants without HCV, suggesting that HCV-mediated chronic inflammation might contribute to the structural bone deficits in this group. Future studies should determine if cure of chronic HCV infection with direct-acting antiviral therapy reverses or prevents the development of bone deficits.

## Supplementary Material

ofaf102_Supplementary_Data

## Supplementary Data

Supplementary materials are available at *Open Forum Infectious Diseases* online. Consisting of data provided by the authors to benefit the reader, the posted materials are not copyedited and are the sole responsibility of the authors, so questions or comments should be addressed to the corresponding author.

## Notes

***Acknowledgments***. We thank the study participants for their participation and the providers at the University of Pennsylvania Health System and Philadelphia FIGHT for their assistance in recruitment. We also thank Delitza Hernandez-Diaz and Nicole LaRatta for their assistance in conducting study visits; Dr Kimberly Forde for her assistance in study design; and the staff of the Nutrition and Growth Lab (Eileen Ford, Gail Jackson, Deborah Ellerbe, Adaobi Obiakor, Regan Murray, David Weber), Research Nursing Core (Audrey Kamrin, Lizzy Sanchez), and Translational Core Laboratory (Nkecha Hughes, Courtney Schmidt, Peace Ani, Sarah Ellor) of the Children's Hospital of Philadelphia's Center for Human Phenomic Science.

***Author contributions*.** V. L. R. had full access to all the study data and takes responsibility for the integrity of the data and the accuracy of the data analysis. Concept and design: V. L. R., M. B. L., X. S. L., D. M. C., C. W. N., J. T. Acquisition, analysis, or interpretation of data: all authors. Drafting of the manuscript: E. J. W., V. L. R., D. M. C., C. W. N. Critical revision of the manuscript for important intellectual content: all authors. Statistical analysis: C. W. N., R. A. H. Obtained funding: V. L. R. Administrative, technical, or material support: D. M. C., S. M. S., K. L. B., J. R. K., S. T., X. S. L., B. S. Z. Supervision: V. L. R., B. S. Z.

***Data availability*.** Data are not publicly available.

***Disclaimer.*** The funding sources had no role in the conduct of the research and/or preparation of the manuscript.

***Financial support*.** This work was supported by the National Institute of Allergy and Infectious Diseases (R01 AI136626 to V. L. R.; F32 AI164809 to E. J. W.) and the National Center for Advancing Translational Sciences (UL1 TR001878) at the National Institutes of Health.
