# Supplementary material for: Abnormal Trabecular and Cortical Bone Microarchitecture in Chronic Hepatitis C Infection and Associations With Select Inflammatory Cytokines
Source: Open Forum Infect Dis. 2025 Apr 29;12(5):ofaf102. doi: 10.1093/ofid/ofaf102 (PMC12039487; doi:10.1093/ofid/ofaf102)
Supplement: ofaf102_Supplementary_Data [file ofaf102_supplementary_data.docx]

**Supplementary Appendix**

**Abnormal Trabecular and Cortical Bone Microarchitecture in Chronic Hepatitis C Infection and Associations With Select Inflammatory Cytokines**

Erica J. Weinstein, Dean M. Carbonari, Craig W. Newcomb, Jessie Torgersen, Shanae M. Smith, Katherine L. Brecker, X. Sherry Liu, Jay R. Kostman, Stacey Trooskin, Rebecca A. Hubbard, Joshua F. Baker, Babette S. Zemel, Mary B. Leonard, Vincent Lo Re III

**Table of Contents**

**Supplementary Methods.** Participant Screening Questions……………………………………………2

**Supplemental Table 1.** Mean differences (95% confidence intervals) in bone measurements between participants with and without hepatitis C virus infection, replacing visceral fat area with fat mass index…………………………………………………………….……………………………………….4

**Supplemental Table 2.** Mean differences (95% confidence intervals) in bone measurements between participants with and without hepatitis C virus infection, after additional adjustment for TNF-alpha levels……………………………………………………...……………………...……...……….6

**Supplemental Table 3.** Mean differences (95% confidence intervals) in log levels of cytokines between participants with and without hepatitis C virus infection, replacing visceral fat area with fat mass index…………………………………………………………….……………………………...……….8

**Supplemental Table 4.** Adjusted mean differences (95% confidence intervals) in HR-pQCT and DXA bone measurements per 1.0 log increase in specified cytokine level, replacing visceral fat area with fat mass index ……………………………………………………………..………………..………..…9

**Supplemental Figure 1**. Directed acyclic graphs for hypothesized exposures, potential confounding variables, and outcomes……………………………………….…………………….……..11

**Supplementary Methods 1.** Participant Screening Questions

1. Are you 18 years of age or older?

▪ No (Not Eligible)

▪ Yes

2. How much do you currently weigh (pounds)?

3. Are you currently pregnant or breastfeeding or plan to become pregnant over the next 18 months?

▪ No/Not Applicable

▪ Yes (Not Eligible)

4. Have you ever had or been diagnosed with chronic kidney disease?

▪ No

▪ Yes (Not Eligible)

5. Have you ever had or been diagnosed with hepatitis B virus infection (HBV)?

▪ No

▪ Yes (Not Eligible)

6. Have you ever had or been diagnosed with celiac disease?

▪ No

▪ Yes (Not Eligible)

7. Have you ever had or been diagnosed with small bowel resection surgery?

▪ No

▪ Yes (Not Eligible)

8. Have you ever had or been diagnosed with chronic diarrhea?

▪ No

▪ Yes (Not Eligible)

9. Have you had weight loss greater than 5% of your body weight within the past 3 months?

▪ No

▪ Yes (Not Eligible)

10. Have you ever had or been diagnosed with cancer?

▪ No

▪ Yes

a) What type or types of cancer have you ever been diagnosed with?

○ Non-melanoma skin cancer ONLY

○ All other cancers (Not Eligible)

11. Have you ever been diagnosed with HIV?

▪ No

▪ Yes

a) Are you currently on an antiretroviral (ART) regimen?

○ No (Not Eligible)

○ Yes

b) Is your HIV viral load undetectable?

○ No (Not Eligible)

○ Yes

12. Have you ever been diagnosed with hepatitis C (HCV)?

▪ No

▪ Yes

a) Have you ever received treatment for your HCV with oral direct acting antiviral

(DAA) therapy?

○ No

○ Yes (Not Eligible)

b) Does your provider plan on treating your HCV with oral direct acting antiviral

(DAA) therapy?

○ No (Not Eligible)

○ Yes

○ Don't Know

# **Supplemental Table 1**. Mean differences (95% confidence intervals) in bone measurements between participants with and without hepatitis C virus infection, replacing visceral fat area with fat mass index. Significant values are bolded.

|  | **Unadjusted** | | **Age-/Sex-/ALMI-/FMI-/Current Smoking-Adjusted** | |
| --- | --- | --- | --- | --- |
| **Bone Measurement** | **Mean Difference**  **(95% CI)** | ***P* Value** | **Mean Difference**  **(95% CI)** | ***P* Value** |
| **HR-pQCT** |  |  |  |  |
| **Radius** |  |  |  |  |
| Trabecular vBMD (mg HA/cm^3^)^*^ | **-25.7 (-42.4, -9.06)** | **0.003** | **-24.0 (-42.1, -5.97)** | **0.010** |
| Total area (mm^2^)^†^ | **12.98 (4.298, 21.66)** | **0.004** | 5.392 (-2.92, 13.70) | 0.201 |
| Cortical vBMD (mg HA/cm^3^)^†^ | **-16.0 (-27.5, -4.51)** | **0.007** | -7.69 (-20.6, 5.238) | 0.241 |
| Cortical area (mm^2^)^†^ | 4.532 (-2.27, 11.33) | 0.189 | 0.426 (-5.30, 6.158) | 0.883 |
| Cortical porosity (%)^†^ | **0.194 (0.025, 0.364)** | **0.025** | 0.094 (-0.059, 0.246) | 0.227 |
| Mean cortical perimeter (mm)^†^ | 1.768 (-0.445, 3.981) | 0.116 | 0.686 (-1.68, 3.057) | 0.567 |
| Mean cortical thickness (mm)^†^ | -0.110 (-0.296, 0.075) | 0.242 | -0.111 (-0.287, 0.064) | 0.210 |
| Mean cortical pore diameter (mm)^†^ | 0.008 (-0.004, 0.020) | 0.184 | 0.001 (-0.012, 0.014) | 0.895 |
| **Tibia** |  |  |  |  |
| Trabecular vBMD (mg HA/cm^3^)^*^ | **-26.5 (-43.6, -9.35)** | **0.003** | **-20.7 (-40.6, -0.762)** | **0.042** |
| Total area (mm^2^)^†^ | 3.825 (-22.8, 30.47) | 0.776 | -6.50 (-32.4, 19.39) | 0.619 |
| Cortical vBMD (mg HA/cm^3^)^†^ | -4.92 (-18.5, 8.694) | 0.475 | -1.34 (-17.6, 14.89) | 0.870 |
| Cortical area (mm^2^)^†^ | -10.4 (-32.1, 11.37) | 0.346 | **-19.1 (-36.1, -2.01)** | **0.029** |
| Cortical porosity (%)^†^ | 0.086 (-0.192, 0.364) | 0.540 | 0.012 (-0.304, 0.327) | 0.942 |
| Mean cortical perimeter (mm)^†^ | 0.380 (-2.37, 3.135) | 0.785 | -0.748 (-3.33, 1.835) | 0.567 |
| Mean cortical thickness (mm)^†^ | -0.336 (-0.740, 0.068) | 0.102 | **-0.474 (-0.812, -0.136)** | **0.007** |
| Mean cortical pore diameter (mm)^†^ | -0.018 (-0.038, 0.003) | 0.086 | -0.017 (-0.040, 0.007) | 0.166 |
| **DXA^‡^** |  |  |  |  |
| Hip BMD (g/cm^2^) | -0.070 (-0.145, 0.005) | 0.069 | -0.031 (-0.111, 0.048) | 0.432 |
| Femoral neck BMD (g/cm^2^) | -0.045 (-0.123, 0.033) | 0.256 | -0.007 (-0.093, 0.079) | 0.871 |
| Lumbar spine BMD (g/cm^2^) | -0.038 (-0.129, 0.052) | 0.400 | -0.027 (-0.128, 0.074) | 0.598 |
| Trabecular bone score | -0.034 (-0.092, 0.024) | 0.244 | -0.003 (-0.064, 0.058) | 0.912 |

Abbreviations: ALMI=appendicular lean mass index; BMD=bone mineral density; DXA=dual-energy x-ray absorptiometry; FMI=fat mass index; HR-pQCT= high-resolution peripheral quantitative computed tomography; vBMD=volumetric bone mineral density

^*^ Measurement at the ultradistal location.

^†^ Measurement at the midshaft location.

^‡^ All analyses additionally adjusted for DXA machine since the machine was changed during the course of the study.

# **Supplemental Table 2**. Mean differences (95% confidence intervals) in bone measurements between participants with and without hepatitis C virus infection, adjusted for specified covariates, before and after additional adjustment for tumor necrosis factor-alpha level . Significant values are bolded.

|  | **Age-/Sex-/ALMI-/Visceral Fat Area-/ Current Smoking-Adjusted** | | **Age-/Sex-/ALMI-/Visceral Fat Area-/ Current Smoking-/TNF-α-Adjusted** | |
| --- | --- | --- | --- | --- |
| **Bone Measurement** | **Mean Difference**  **(95% CI)** | ***P* Value** | **Mean Difference**  **(95% CI)** | ***P* Value** |
| **HR-pQCT** |  |  |  |  |
| **Radius** |  |  |  |  |
| Trabecular vBMD (mg HA/cm^3^)^*^ | **-24.2 (-42.7, -5.72)** | **0.011** | -11.6 (-33.3, 10.20) | 0.2938 |
| Total area (mm^2^)^†^ | 3.977 (-4.61, 12.57) | 0.360 | 4.001 (-5.98, 13.98) | 0.4275 |
| Cortical vBMD (mg HA/cm^3^)^†^ | -8.32 (-21.6, 4.975) | 0.217 | -6.34 (-20.5, 7.804) | 0.3756 |
| Cortical area (mm^2^)^†^ | -0.662 (-6.56, 5.234) | 0.824 | 0.995 (-5.77, 7.760) | 0.7706 |
| Cortical porosity (%)^†^ | 0.101 (-0.057, 0.258) | 0.207 | 0.083 (-0.097, 0.263) | 0.3613 |
| Mean cortical perimeter (mm)^†^ | 0.734 (-1.73, 3.202) | 0.556 | 0.270 (-2.61, 3.149) | 0.8524 |
| Mean cortical thickness (mm)^†^ | -0.124 (-0.304, 0.057) | 0.177 | -0.054 (-0.255, 0.147) | 0.5947 |
| Mean cortical pore diameter (mm)^†^ | 0.001 (-0.013, 0.014) | 0.933 | -0.002 (-0.017, 0.013) | 0.7966 |
| **Tibia** |  |  |  |  |
| Trabecular vBMD (mg HA/cm^3^)^*^ | **-20.5 (-40.9, -0.078)** | **0.049** | -12.7 (-35.6, 10.11) | 0.2705 |
| Total area (mm^2^)^†^ | -10.3 (-37.1, 16.44) | 0.446 | -22.1 (-52.7, 8.491) | 0.1543 |
| Cortical vBMD (mg HA/cm^3^)^†^ | -2.19 (-18.8, 14.42) | 0.794 | 11.14 (-5.46, 27.75) | 0.1855 |
| Cortical area (mm^2^)^†^ | **-20.9 (-38.3, -3.48)** | **0.019** | **-23.0 (-43.6, -2.48)** | **0.0286** |
| Cortical porosity (%)^†^ | 0.051 (-0.270, 0.372) | 0.753 | -0.136 (-0.470, 0.198) | 0.4213 |
| Mean cortical perimeter (mm)^†^ | -1.02 (-3.69, 1.641) | 0.447 | -2.49 (-5.48, 0.506) | 0.1021 |
| Mean cortical thickness (mm)^†^ | **-0.471 (-0.818, -0.125)** | **0.008** | **-0.418 (-0.816, -0.020)** | **0.0400** |
| Mean cortical pore diameter (mm)^†^ | -0.013 (-0.037, 0.011) | 0.277 | -0.013 (-0.041, 0.015) | 0.3559 |
| **DXA^‡^** |  |  |  |  |
| Total hip BMD (g/cm^2^) | -0.028 (-0.109, 0.053) | 0.492 | -0.030 (-0.122, 0.062) | 0.5182 |
| Femoral neck BMD (g/cm^2^) | -0.011 (-0.098, 0.076) | 0.800 | -0.005 (-0.104, 0.094) | 0.9235 |
| Lumbar spine BMD (g/cm^2^) | -0.024 (-0.128, 0.079) | 0.641 | -0.013 (-0.131, 0.104) | 0.8256 |
| Trabecular bone score | -0.020 (-0.080, 0.039) | 0.496 | 0.000 (-0.066, 0.067) | 0.9911 |

Abbreviations: ALMI=appendicular lean mass index; BMD=bone mineral density; DXA=dual-energy x-ray absorptiometry; FMI=fat mass index; HR-pQCT= high-resolution peripheral quantitative computed tomography; TNF=tumor necrosis factor; vBMD=volumetric bone mineral density

^*^ Measurement at the ultradistal location.

^†^ Measurement at the midshaft location.

^‡^ All analyses additionally adjusted for DXA machine since the machine was changed during the course of the study.

# **Supplemental Table 3.** Mean differences (95% confidence intervals) in log levels of cytokines between participants with and without hepatitis C virus infection, adjusted for age, sex assigned at birth, fat mass index, and current smoking status. Significant values are bolded.

|  | **Unadjusted** | | **Age-/Sex-/FMI-/Current Smoking-Adjusted** | | |
| --- | --- | --- | --- | --- | --- |
| **Cytokine** | **Mean Difference**  **(95% CI)** | ***P* Value** | **Mean Difference**  **(95% CI)** | ***P* Value** |  |
| Log Interleukin-6 | 0.134 (-0.012, 0.280) | 0.072 | 0.132 (-0.017, 0.280) | 0.082 |  |
| Log Interleukin-18 | **0.138 (0.051, 0.224)** | **0.002** | 0.058 (-0.041, 0.157) | 0.249 |  |
| Log TNF-α | **0.114 (0.069, 0.158)** | **<0.001** | **0.091 (0.038, 0.144)** | **0.001** |  |

Abbreviations: CI=confidence interval; FMI=fat mass index; TNF-α=tumor necrosis factor-α

# **Supplemental Table 4.** Adjusted mean differences (95% confidence intervals) in HR-pQCT and DXA bone measurements per 1.0 log increase in specified cytokine level. All outcomes are adjusted for age, sex assigned at birth, appendicular lean mass index, fat mass index, current smoking status, and hepatitis C infection. Significant values are bolded.

| **Bone Measurement** | **Mean Difference^*^ (95% CI) in Specified Bone Measurement**  **per 1.0 Log Increase in Specified Cytokine** | | | | | |
| --- | --- | --- | --- | --- | --- | --- |
|  | **Log IL-6** | ***P* Value** | **Log IL-18** | ***P* Value** | **Log TNF-α** | ***P* Value** |
| **HR-pQCT** |  |  |  |  |  |  |
| **Radius** |  |  |  |  |  |  |
| Trabecular vBMD (mg HA/cm^3^)^†^ | 13.23 (-14.9, 41.31) | 0.352 | **-41.1 (-81.8, -0.458)** | **0.048** | **-97.4 (-176, -19.0)** | **0.016** |
| Total area (mm^2^)^‡^ | 2.866 (-9.63, 15.37) | 0.650 | -6.41 (-24.9, 12.09) | 0.493 | -2.31 (-38.0, 33.39) | 0.898 |
| Cortical vBMD (mg HA/cm^3^)^‡^ | 3.844 (-13.9, 21.63) | 0.669 | 6.532 (-20.0, 33.08) | 0.626 | 2.040 (-48.8, 52.90) | 0.937 |
| Cortical area (mm^2^)^‡^ | 4.643 (-3.81, 13.10) | 0.278 | -8.97 (-21.4, 3.498) | 0.156 | -4.15 (-28.4, 20.13) | 0.735 |
| Cortical porosity (%)^‡^ | 0.120 (-0.107, 0.347) | 0.296 | -0.102 (-0.440, 0.236) | 0.550 | 0.405 (-0.241, 1.050) | 0.216 |
| Mean cortical perimeter (mm)^‡^ | 1.057 (-2.52, 4.634) | 0.559 | -1.20 (-6.51, 4.105) | 0.654 | 3.299 (-6.94, 13.54) | 0.524 |
| Mean cortical thickness (mm)^‡^ | 0.244 (-0.003, 0.491) | 0.053 | -0.190 (-0.562, 0.182) | 0.312 | -0.008 (-0.731, 0.715) | 0.983 |
| Mean cortical pore diameter (mm)^‡^ | 0.014 (-0.006, 0.033) | 0.162 | -0.009 (-0.038, 0.020) | 0.546 | 0.040 (-0.016, 0.095) | 0.157 |
| **Tibia** |  |  |  |  |  |  |
| Trabecular vBMD (mg HA/cm^3^)^†^ | 9.534 (-20.0, 39.09) | 0.523 | -40.9 (-82.9, 1.023) | 0.056 | -50.8 (-134, 32.46) | 0.228 |
| Total area (mm^2^)^‡^ | 23.18 (-15.3, 61.63) | 0.234 | 5.547 (-49.9, 60.96) | 0.843 | 13.58 (-96.8, 123.9) | 0.807 |
| Cortical vBMD (mg HA/cm^3^)^‡^ | -10.6 (-32.7, 11.55) | 0.345 | -18.3 (-50.1, 13.53) | 0.256 | **-89.9 (-150, -29.7)** | **0.004** |
| Cortical area (mm^2^)^‡^ | 5.776 (-20.4, 31.99) | 0.662 | 3.586 (-34.2, 41.36) | 0.851 | -23.7 (-98.6, 51.17) | 0.530 |
| Cortical porosity (%)^‡^ | **0.527 (0.097, 0.957)** | **0.017** | 0.319 (-0.315, 0.952) | 0.320 | **1.474 (0.256, 2.692)** | **0.018** |
| Mean cortical perimeter (mm)^‡^ | 2.592 (-1.17, 6.355) | 0.174 | 2.518 (-2.91, 7.945) | 0.359 | 3.879 (-6.93, 14.69) | 0.477 |
| Mean cortical thickness (mm)^‡^ | -0.175 (-0.679, 0.328) | 0.490 | -0.028 (-0.759, 0.702) | 0.939 | -0.744 (-2.19, 0.698) | 0.308 |
| Mean cortical pore diameter (mm)^‡^ | 0.024 (-0.012, 0.060) | 0.192 | 0.008 (-0.045, 0.061) | 0.762 | 0.006 (-0.098, 0.109) | 0.915 |
| **DXA^¶^** |  |  |  |  |  |  |
| Total hip BMD (g/cm^2^) | 0.079 (-0.023, 0.182) | 0.128 | -0.059 (-0.210, 0.093) | 0.446 | -0.257 (-0.550, 0.035) | 0.084 |
| Femoral neck BMD (g/cm^2^) | **0.117 (0.008, 0.226)** | **0.036** | -0.069 (-0.232, 0.095) | 0.406 | -0.295 (-0.610, 0.020) | 0.066 |
| Lumbar spine BMD (g/cm^2^) | 0.082 (-0.046, 0.211) | 0.207 | -0.061 (-0.258, 0.135) | 0.535 | -0.306 (-0.676, 0.064) | 0.104 |
| Trabecular bone score | **-0.089 (-0.163, -0.014)** | **0.020** | -0.044 (-0.159, 0.070) | 0.445 | **-0.276 (-0.493, -0.060)** | **0.013** |

Abbreviations: BMD=bone mineral density; IL=interleukin; DXA=dual-energy X-ray absorptiometry; HCV=hepatitis C virus; HR-pQCT=high-resolution peripheral quantitative computed tomography; TNF=tumor necrosis factor; vBMD=volumetric bone mineral density

^*^ Mean difference was adjusted for age, sex assigned at birth, appendicular lean mass index, fat mass index, current smoking, and chronic HCV infection.

^†^ Measurement at the ultradistal location.

^‡^ Measurement at the midshaft location.

^¶^ Analyses additionally adjusted for DXA machine since the machine was changed during the course of the study.

**Supplementary Figure 1.** Directed acyclic graphs for hypothesized exposures, potential confounding variables, and outcomes. The purple boxes indicate potential confounding variables, green boxes indicate mediator variables, and the white box indicates a collider in the relationships between exposures (at left) and outcomes (at right), designated by blue boxes. Orange boxes indicate the potential pathways by which exposure could contribute to the outcome.

1. Relation between chronic hepatitis C virus infection and abnormal bone.


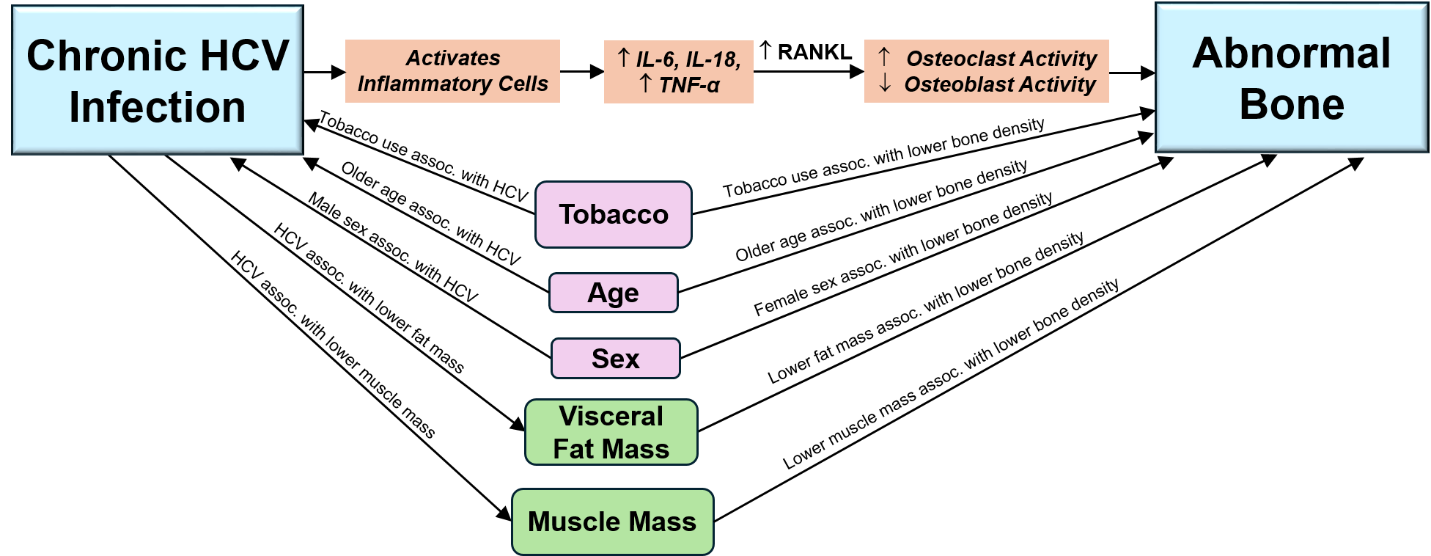


1. Relation between chronic hepatitis C virus infection and inflammatory cytokines.


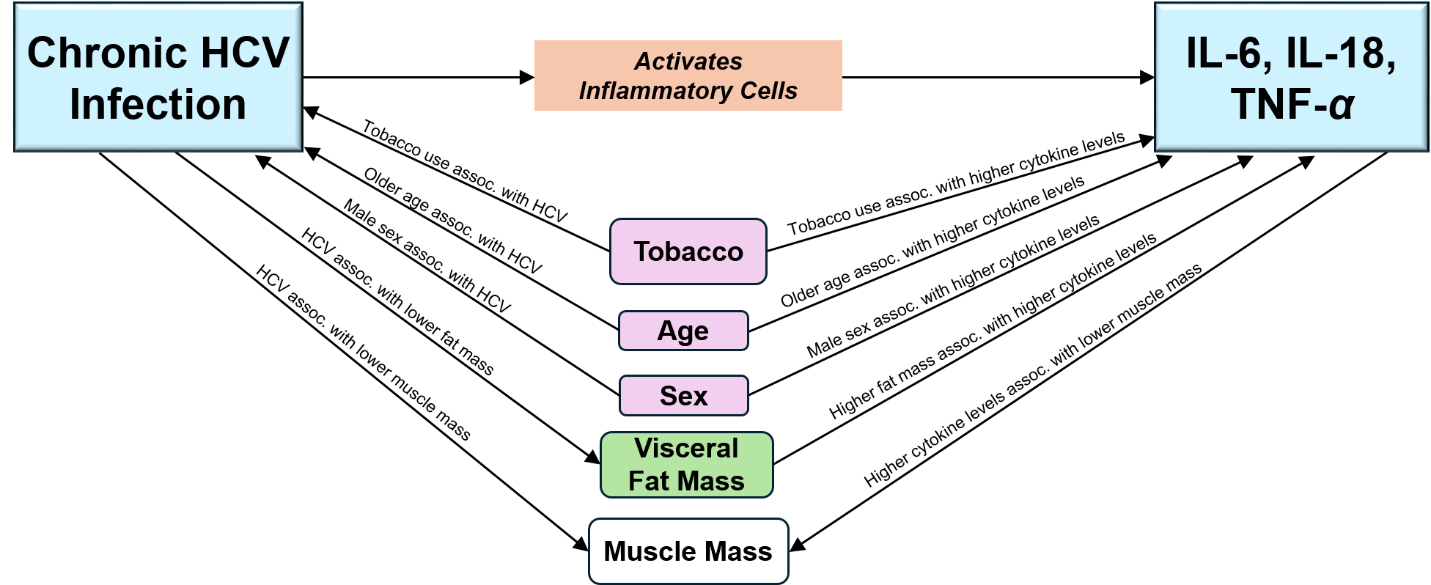


1. Relation between inflammatory cytokines and abnormal bone.


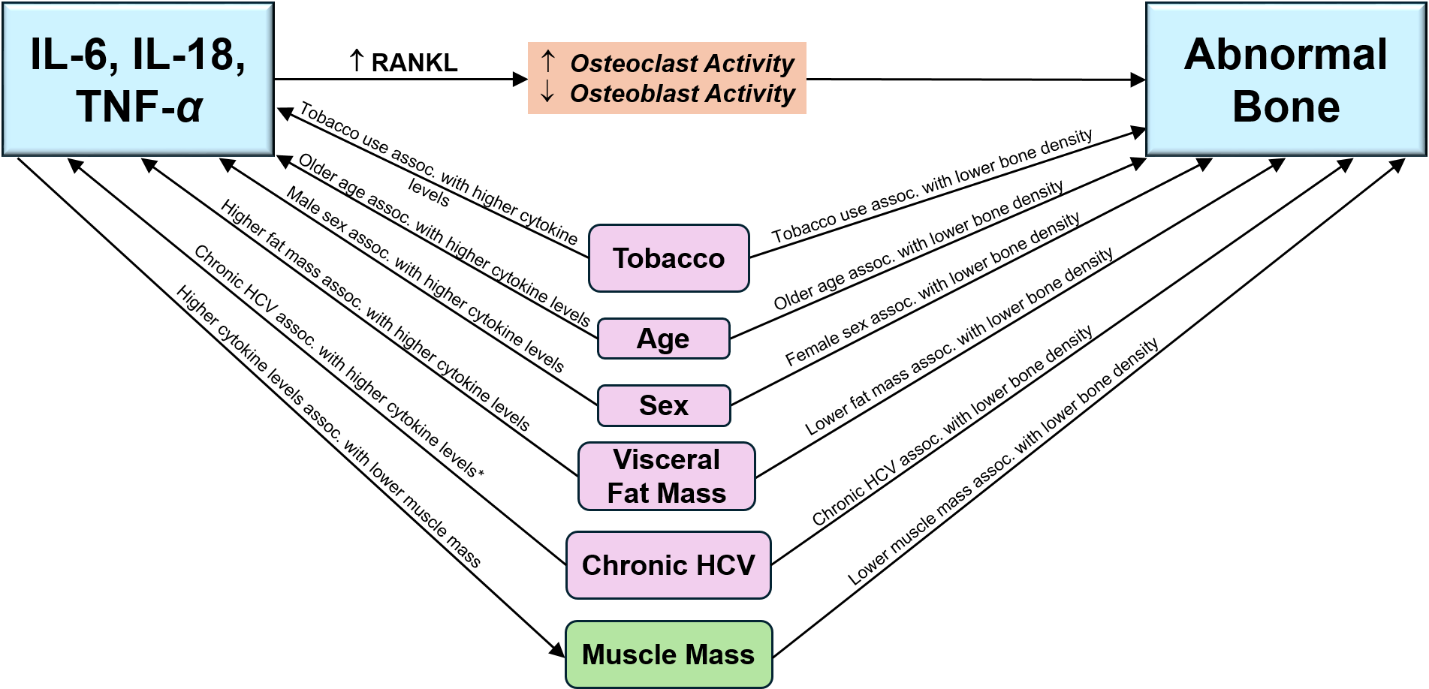


Abbreviations: assoc.=associated; HCV=hepatitis C virus; IL-6=interleukin-6; IL-18=interleukin-18; RANKL=receptor activator of nuclear factor kappa beta ligand; TNF-α=tumor necrosis factor-α

^*^ Refer to Panel B for additional relationships between chronic HCV and inflammatory cytokines.
